# Maximal skin wettedness as a function of environment and metabolic rate in unacclimated young and older adults (PSU HEAT Project)

**DOI:** 10.1007/s00484-025-02986-5

**Published:** 2025-07-16

**Authors:** Kat G. Fisher, Olivia K. Leach, Rachel M. Cottle, W. Larry Kenney

**Affiliations:** 1 Department of Kinesiology, Pennsylvania State University, University Park, PA 16802 USA; 2 Graduate Program in Physiology, Pennsylvania State University, University Park, PA 16802 USA

**Keywords:** Aging, Sweat evaporation, Heat balance, Critical environmental limits, Thermoregulation

## Abstract

Maximal skin wettedness (ω_max_), the proportion of the skin covered in sweat at the upper limits of compensable heat stress, is an important parameter for modeling human heat stress responses. We previously determined ω_max_ in two extreme environments during activities of daily living across the life span; however, ω_max_ has yet to be quantified across a broader range of environments and metabolic rates for young (Y) and older (O) adults in extreme heat. The present study used partitional calorimetry to determine ω_max_ across a wide range of hot environments (34–49 °C dry-bulb temperature, T_db_; 14–80% relative humidity, rh) in 51 Y (18–35 yrs; 29 F) and 55 O (65–92 yrs; 33 F) during minimal activity (MinAct; ~150 W), light ambulation (LightAmb; ~250 W; Y only), and rest (O only; ~90 W). During MinAct, ω_max_ was higher in Y compared to O across environments (all *P* ≤ 0.008) and ranged from 0.43 to 0.99 as humidity increased in Y and 0.21 to 0.83 in O. During LightAmb in Y, ω_max_ ranged from 0.53 to 1.10 but was higher compared to MinAct only in hot-dry environments (*P* < 0.0001). At rest in O, ω_max_ ranged from 0.16 to 0.78 and was lower compared to MinAct only in a 53–60% rh condition (36 °C, T_db_) (*P* < 0.008). These findings indicate that ω_max_ varies with age, metabolic rate, and environment. ω_max_ established herein for unacclimated young and older adults across environments and relatively low metabolic rates can be used for heat stress modeling in these populations and environments.

## Introduction

The primary mechanism by which the human body is cooled during passive and exertional heat stress is the evaporation of sweat. Maximal evaporative heat loss is directly related to the proportion of the body covered in sweat, i.e., maximal skin wettedness (ω_max_). Current heat strain standards, such as ISO 7933, use ω_max_ to calculate heat exchange between an individual and their environment, but rely on a previously determined ω_max_ of 1.00 for acclimated individuals and 0.85 for unacclimated individuals (Candas et al. 1979). However, these ω_max_ values are derived from of a small sample size of young adult men lying supine. More recently, Ravanelli et al. determined ω_max_ in unacclimated, untrained adults to be ~ 0.72 (Ravanelli et al. 2018). However, this is specific to a warm-humid environment and a metabolic heat production of 450 W, a much higher metabolic rate than in the present study.

Using partitional calorimetry in conjunction with a progressive heat stress protocol, our laboratory previously determined ω_max_ in young, middle-aged, and older adults in a single hot-dry and single -warm-humid environment at a low metabolic rate representative of activities of daily living (Fisher et al. 2024). Young unacclimated adults exhibited mean ω_max_ of 0.69 and 0.52 in the warm-humid and hot-dry environments, respectively. Older unacclimated adults exhibited significantly lower ω_max_ of 0.47 and 0.40 in the warm-humid and hot-dry environments. ω_max_ was further reduced when the older adults were resting, illustrating the variability present in ω_max_ as a function of age, environmental condition, and the intensity of the activity preformed.

Previous work in our laboratory empirically derived critical environmental limits representing the boundary between compensable and uncompensable heat stress across the adult age span from 18 to 92 yrs (Wolf et al. 2022, 2023; Cottle and Fisher 2024). These data represent a best-case scenario for prolonged exposure in an indoor setting during seated rest or at low metabolic rates representing activities of daily living in and around the home and illustrate the decline in critical environmental limits with advancing age. As ω_max_ represents the highest achievable skin wettedness at the critical environmental limit, establishing empirically derived ω_max_ for specific age cohorts, metabolic rates, and environmental conditions can aid in more accurate heat stress modeling and prediction.

Therefore, the purpose of the present study was to use partitional calorimetry to determine ω_max_ at the previously established critical environmental limits (Wolf et al. 2022, 2023) (i.e., the upper limit of compensable heat stress) for young and older adults across a wider spectrum of environmental conditions and at multiple low metabolic rates.

## Methods

### Subjects

All testing was conducted in controllable environmental chambers housed in Noll Laboratory at the Pennsylvania State University and all procedures were approved by the Pennsylvania State University Institutional Review Board and conformed to the Declaration of Helsinki’s stated guidelines. Prior to participation participants gave oral and written consent after being informed of all aspects of the experimental study. All experimental procedures are registered on ClinicalTrials.gov (NCT0428439).

Calculated values for ω_max_ presented herein utilized data collected at the upper limits of compensable heat stress that were previously determined for the present cohort of subjects (Wolf et al. 2022, 2023). Young and older groups were aged 18–29 yrs and 65–89 yrs, respectively. To increase generalizability of the data, subjects were recruited without regard to individual characteristics such as body size, aerobic fitness, blood pressure, etc. There was no attempt to control for menstrual status, contraceptive use, or heat acclimatization status. Before each experiment, subjects refrained from vigorous exercise for 24 h and caffeine for 12 h and provided a urine sample upon arrival to ensure euhydration (urine specific gravity ≤ 1.020) (PAL-S, Atago, Bellevue, WA, USA). All participants wore standard clothing during experiments of ~ 0.3 CLO, consisting of a short-sleeved t-shirt, a sports bra (women), athletic shorts, socks, and walking/running shoes.

### Experimental procedures

All experimental procedures have been previously reported in detail (Wolf et al. 2022, 2023). The a prior power analysis for the previous research applies here (Wolf et al. 2023). All subjects performed light physical activity on a cycle ergometer against zero resistance at a cadence of 40–50 rpm to represent the metabolic cost associated with minimal activities of daily living (MinAct; ~150 W). Older subjects completed additional experimental trials during seated rest (~ 90 W). Young subjects completed additional trials walking continuously on a motor-driven treadmill at a speed of 2.2 mi/h and grade of 3% to achieve light ambulatory activity (LightAmb; ~250 W).

During critical water vapor pressure trials (P_crit_), dry-bulb temperature (T_db_) was held constant at either 34, 36, 38, or 40 °C. During critical temperature trials (T_crit_), water vapor pressure (P_a_) was held constant at either 12, 16, or 20 mmHg. Following a 30-minute equilibration period in which ambient chamber conditions were held constant, either T_db_ (T_crit_ tests) or P_a_ (P_crit_ tests) were increased in a stepwise fashion (1 °C or 1 mmHg every five minutes) until a clear upward inflection in T_c_ was detected. Experimental trials lasted approximately 90–120 min. The T_c_ inflection marked the upper limit of compensable heat stress for that trial. ω_max_ was calculated using environmental and physiological data at the T_c_ inflection point.

### Measurements and partitional calorimetry calculations

Continuous measurements of T_c_ were recorded using gastrointestinal temperature telemetry capsules (VitalSense, Philips Respironics, Bend, OR, USA; BodyCap, Hérouville-Saint-Clair, France). Subjects ingested the capsules 1–2 h before reporting to the laboratory in accordance with previously published data demonstrating that ingestion times from 1 to 12 h before use do not influence the precision of T_c_ data (Notley et al. 2021). Skin temperature was measured continuously on each subject’s chest (T_chest_), arm (T_arm_), thigh (T_thigh_), and lower leg (T_leg_) and was used to calculate a weighted mean skin temperature (T_sk_):

1 $$\:Tsk=0.3\left(Tchest\right)+0.3\left(Tthigh\right)+0.2\left(Tarm\right)+0.2\left(Tleg\right)\:[^\circ\:C]$$

To calculate thermal balance parameters and ω_max_, partitional calorimetry was used. The rate of oxygen uptake (VO_2_) and respiratory exchange ratio (RER) were measured twice during experimental trials at 5 and 60 min using open-circuit spirometry (Parvo Medics TrueOne^®^ 2400, Parvo, UT, USA). Average VO_2_ and RER values from the two time points (which did not differ significantly), were used to calculate metabolic rate. Metabolic rate [M; Watts (W)], normalized to body surface area, was calculated from V̇o_2_ and RER (Cramer and Jay 2019) as:

2 $$\begin{aligned}&M={\dot{V}o}_{2}\bullet\\&\frac{\left[\left(\left(\frac{RER-0.7}{0.3}\right)\bullet\:21.13\right)+\left(\left(\frac{1.0-RER}{0.3}\right)\bullet\:19.62\right)\right]}{60}\bullet\:\text{1,000}\bullet\:{{A}_{D}}^{-1}\end{aligned}$$

where A_D_ is the Dubois surface area (m^2^). Because minimal external work (W) was done by participants during MinAct and rest trials, M equaled net metabolic rate (M_net_). For treadmill walking trials, external work was calculated as

3 $$\:W=9.81\bullet\:{m}_{b}\bullet\:{v}_{w}\bullet\:{F}_{g}\bullet\:{{A}_{D}}^{-1}$$

where m_b_ is the body mass (k), V_w_ is the walking velocity, and F_g_ is the fractional grade of the treadmill (Cramer and Jay 2019). For walking M_net_ was calculated as *M – W.*

Heat balance calculations were performed at the final compensable combination of T_db_ and P_a_, i.e., the 5-min period immediately preceding the upward core temperature inflection. Dry heat exchange via radiation and convection (R + C; W∙m^−2^) was determined (Cramer and Jay 2019; Barker et al. 1999) as

4 $$\:\left(R+C\right)=\frac{{(\text{T}}_{\text{d}\text{b}}-{\stackrel{-}{\text{T}}}_{\text{s}\text{k}})}{{I}_{T}}\:$$

where $\:{I}_{T}$ is assumed to be 0.163 m^2^∙°C∙W^−1^ and is the total calculated insulation between the skin surface to the environment (Barker et al. 1999). In conditions of greater air or body movement $\:{I}_{T}$ should be adjusted to a resultant value via ISO9920 to reflect the air movement.

Maximal evaporative capacity of the environment (E_max_; W∙m^−2^) was calculated as (Barker et al. 1999)

5 $$\:{E}_{\text{m}\text{a}\text{x}}=\frac{{(\text{P}}_{\text{s},\text{s}\text{k}}-{P}_{\text{a}})}{{R}_{e,t}}$$

where P_s, sk_ is the saturated vapor pressure at the skin surface (assuming 100% relative humidity at the given skin temperature) and $\:{R}_{e,t}$ the clothing total evaporative resistance, is 0.13 m^2^∙mmHg∙W^−1^ (Wang 2011).

The evaporative cooling required to maintain thermal balance (E_req_; W∙m^−2^) was calculated from the heat balance equation as:

6 $$\:{E}_{req}={M}_{\text{n}\text{e}\text{t}}+\left(R+C\right)+{C}_{res}-{E}_{res}-\:S$$

where C_res_ (the convective heat loss associated with respiration; <1 W∙m^−2^), E_res_ (evaporative heat loss associated with respiration; ≤2 W∙m^−2^), and S (heat storage) were considered to be negligible (Cottle et al. 2022).

Maximum skin wettedness (ω_max_; unitless) was calculated from E_req_ and E_max_ (Cramer and Jay 2019) as

7 $$\:{{\upomega\:}}_{max}=\frac{{E}_{req}}{{E}_{max}}$$

Total sweat rate (SR, g∙m^−2^∙h^−1^) and percent body mass loss (% BML) were determined during each experiment from the loss of nude body mass on a scale accurate to ± 10 g. Fluid intake was prohibited between the initial and final measurements of nude body mass.

### Statistical analyses

All statistical analyses were performed using IBM SPSS Statistics, v. 28, IBM Corp., Armonk, NY. Independent samples t-tests were used to compare subject characteristics present in Table 1 between young and older adults. Independent samples t-tests were used to compare ω_max_ in each environmental condition between young and older adults during MinAct. Differences in ω_max_ due to metabolic rate were compared within young and older adults individually using pair-samples t-test. Graphical and Table 1 data are reported as means ± SD. Tables 2 and 3 data is presented as mean and 95% confidence intervals. The Bonferroni method was used to account for multiple comparisons between age groups and metabolic rates, significance was accepted at α = 0.008 for the age group comparison and older adults (6 comparisons) and α = 0.007 for young adults.

**Table 1:** Subject characteristics

Characteristic	Young	Older
n	51 (29 F)	55 (33 F)
Age Range (yr)	18–33	65–92
Height (m)	1.7 ± 0.1	1.7 ± 0.1
Weight (kg)	75 ± 15	73 ± 17
BMI (kg∙m^−2^)	25 ± 4	26 ± 5
A_D_ (m^2^)	1.9 ± 0.2	1.8 ± 0.2
A_D_∙mass^−1^ (m^2^∙kg^−1^)	0.026 ± 0.002	0.025 ± 0.003
VO_2max_ (ml∙kg^−1^∙min^−1^)	46 ± 12	28 ± 9*

*P < 0.001 compared to young. A_D_, DuBois body surface area; A_D_·kg^−1^, body surface area-to-mass ratio; Vo_2max_, maximal oxygen consumption

**Table 2:** Environmental conditions, partitional calorimetry parameters, and ω_max_ for young adults during minact and lightamb in all environments

MinAct	34 °C	36 °C	38 °C	40 °C	12 mmHg	16 mmHg	20 mmHg
T_db_ (°C)	33.9	36.0	38.1	40.1	49.3	46.4	43.7
	(33.7, 34.2)	(35.9, 36.1)	(38.0, 38.3)	(39.9, 40.3)	(48.2, 50.4)	(45.5, 47.3)	(42.5, 44.9)
rh (%)	79.7	66.5	46.9	36.8	13.8	20.7	29.5
	(77.6, 81.7)	(64.1, 69.0)	(45.6, 48.2)	(34.4, 39.1)	(12.9, 14.7)	(19.9, 21.5)	(27.8, 31.2)
T_wb_ (°C)	30.8	30.4	31.1	30.6	25.5	26.9	27.8
	(30.5, 31.9)	(29.9, 30.8)	(30.2, 32.0)	(30.0, 31.3)	(25.3, 25.8)	(26.6, 27.2)	(27.4, 28.2)
P_a_ (mmHg)	31.7	29.6	29.6	27.6	12.2	16.3	19.7
	(30.9, 32.5)	(28.6, 30.7)	(27.7, 31.5)	(26.4, 28.9)	(12.0, 12.4)	(16.0, 16.5)	(19.3, 20.1)
SR (g∙m^−2^∙h^−1^)	122	140	196	178	167	142	121
	(93, 150)	(97, 184)	(143, 249)	(141, 215)	(123, 210)	(109, 174)	(89, 152)
$\:{\stackrel{-}{\text{T}}}_{\text{s}\text{k}}$(°C)	36.2	36.3	36.5	37.7	38.5	38.0	37.5
	(36.1, 36.4)	(36.1, 36.4)	(36.2, 36.8)	(36.4, 37.1)	(38.3, 38.7)	(37.8, 38.2)	(37.1, 37.8)
(R + C) (W∙m^−2^)	−14	−2	10	21	66	52	38
	(−15, −13)	(−3, −1)	(8, 12)	(19, 23)	(60, 72)	(46, 57)	(34, 42)
E_req_ (W∙m^−2^)	70	74	144	156	155	137	122
	(66, 74)	(68, 79)	(138, 151)	(150, 163)	(147, 164)	(130, 144)	(115, 128)
E_max_ (W∙m^−2^)	101	115	169	184	297	259	221
	(94, 107)	(109, 122)	(158, 180)	(171, 198)	(294, 300)	(255, 263)	(216, 226)
ω_max_	0.71	0.65	0.73	0.65	0.52	0.53	0.55
	(0.65, 0.77)	(0.59, 0.70)	(0.68, 0.93)	(0.61, 0.79)	(0.49, 0.55)	(0.50, 0.56)	(0.52, 0.58)
LightAmb	34 °C	36 °C	38 °C	40 °C	12 mmHg	16 mmHg	20 mmHg
T_db_ (°C)	33.8	35.9	38.1	40.0	44.5	42.5	39.1
	(33.7, 34.0)	(35.9, 36.0)	(37.8, 38.3)	(39.9, 40.1)	(43.4, 45.5)	(41.7, 43.2)	(38.5, 39.7)
rh (%)	60.8	54.6	60.9	51.0	18.6	25.6	37.9
	(57.3, 64.4)	(52.5, 56.7)	(56.3, 65.6)	(48.2, 53.8)	(17.1, 20.0)	(24.5, 26.6)	(36.9, 38.8)
T_wb_ (°C)	27.4	28.0	28.2	27.2	24.1	25.7	26.8
	(26.7, 28.0)	(27.6, 28.4)	(27.9, 28.4)	(26.5, 27.8)	(23.8, 24.5)	(25.5, 25.9)	(26.5, 27.0)
P_a_ (mmHg)	24.0	24.2	23.0	20.9	12.1	16.0	20.0
	(22.7, 25.4)	(23.3, 25.2)	(22.4, 23.6)	(19.5, 22.2)	(11.9, 12.3)	(15.9, 16.2)	(19.7, 20.2)
SR (g∙m^−2^∙h^−1^)	179	205	269	291	223	194	197
	(150, 207)	(168, 242)	(220, 317)	(246, 335)	(188, 258)	(153, 235)	(170, 225)
$\:{\stackrel{-}{\text{T}}}_{\text{s}\text{k}}$(°C)	35.9	35.8	36.6	37.0	37.4	37.1	36.0
	(35.7, 36.1)	(35.6, 36.1)	(36.4, 36.8)	(36.8, 37.3)	(37.2, 37.6)	(36.9, 37.3)	(35.6, 36.4)
(R + C) (W∙m^−2^)	−13	0	9	18	38	32	19
	(−15, −11)	(−1, 2)	(8, 10)	(17, 20)	(33, 43)	(28, 36)	(16, 22)
E_req_ (W∙m^−2^)	125	132	88	99	180	163	152
	(117, 133)	(125, 140)	(83, 93)	(94, 103)	(171, 189)	(156, 170)	(144, 160)
E_max_ (W∙m^−2^)	155	151	117	141	279	241	188
	(145, 165)	(145, 156)	(102, 133)	(128, 154)	(273, 285)	(235, 247)	(181, 194)
ω_max_	0.82	0.88	0.84	0.83	0.65	0.68	0.82
	(0.75, 0.88)	(0.82, 0.94)	(0.78, 0.98)	(0.78, 0.97)	(0.62, 0.68)	(0.64, 0.72)	(0.75, 0.88)

Data are presented as MEAN (95% confidence intervals). T_db_, ambient dry-bulb temperature at critical environmental limit; rh, relative humidity at critical environmental limit; T_wb_, ambient wet-bulb temperature at critical environmental limit; P_a_, ambient water vapor pressure at critical environmental limit; ${\overline{\mathrm T}}_{\mathrm{sk}}$, mean skin temperature at critical environmental limit; SR, sweat rate; (R + C), radiative and convective heat exchange; E_req_, evaporative cooling required to maintain thermal balance; E_max_, maximal evaporative heat loss; ω_max_, maximal skin wettedness

**Table 3:** Environmental conditions, partitional calorimetry parameters, and ω_max_ for older adults during rest and minact in all environments

Rest	34 °C	36 °C	38 °C	40 °C	12 mmHg	16 mmHg
T_db_ (°C)	33.9	36.2	38.1	40.1	46.3	43.4
	(33.7, 34.2)	(35.9, 36.4)	(37.9, 38.3)	(40.0, 40.1)	(44.3, 48.2)	(41.1, 45.6)
rh (%)	59.2	60.0	53.4	37.7	15.6	25
	(55.0, 63.4)	(55.6, 64.5)	(48.3, 58.5)	(33.5, 41.9)	(13.7, 17.4)	(22.2, 27.8)
T_wb_ (°C)	26.9	29.2	29.5	27.4	24.4	25.9
	(26.0, 27.7)	(28.3, 30.1)	(28.4, 30.6)	(26.3, 28.5)	(23.9, 24.9)	(25.3, 26.6)
P_a_ (mmHg)	23.5	26.9	26.7	21.6	11.7	16.1
	(21.8, 25.2)	(24.9, 28.9)	(24.2, 29.3)	(19.3, 23.9)	(11.2, 12.2)	(15.7, 16.5)
SR (g∙m^−2^∙h^−1^)	48	51	91	75	58	49
	(35, 61)	(33, 70)	(27, 155)	(40, 111)	(34, 81)	(35, 63)
$\:{\stackrel{-}{\text{T}}}_{\text{s}\text{k}}$(°C)	35.5	36.2	36.4	36.7	37.3	36.8
	(35.2, 35.8)	(36.0, 36.4)	(36.2, 36.6)	(36.4, 37.0)	(36.9, 37.6)	(36.3, 37.3)
(R + C) (W∙m^−2^)	−10	0	11	20	48	37
	(−12, −8)	(−2, 1)	(9, 12)	(18, 23)	(37, 60)	(24, 49)
E_req_ (W∙m^−2^)	39	46	60	72	93	88
	(33, 45)	(42, 50)	(54, 66)	(67, 77)	(78, 108)	(73, 102)
E_max_ (W∙m^−2^)	151	133	137	187	286	232
	(133, 168)	(121, 145)	(118, 156)	(165, 210)	(255, 318)	(220, 244)
ω_max_	0.28	0.36	0.48	0.40	0.33	0.37
	(0.21, 0.35)	(0.31, 0.41)	(0.38, 0.58)	(0.34, 0.46)	(0.28, 0.37)	(0.32, 0.43)
MinAct	34 °C	36 °C	38 °C	40 °C	12 mmHg	16 mmHg
T_db_ (°C)	33.8	36.2	38.0	40.2	42.9	40.4
	(33.6, 34.1)	(36, 36.4)	(37.8, 38.3)	(40.0, 40.3)	(40.9, 44.8)	(38.5, 42.2)
rh (%)	58.4	52.8	46.1	33.7	18.8	31.5
	(48.9, 68.0)	(47.2, 58.4)	(41.7, 50.6)	(28.9, 38.5)	(17.0, 20.5)	(27.6, 35.4)
T_wb_ (°C)	26.8	27.8	27.8	26.4	23.5	24.5
	(24.9, 28.7)	(26.5, 29.0)	(26.7, 29.0)	(25.1, 27.7)	(22.9, 24.2)	(23.8, 25.1)
P_a_ (mmHg)	23.1	24.9	23.0	19.5	11.9	15.8
	(20.0, 26.3)	(22.4, 27.3)	(20.6, 25.5)	(16.8, 22.2)	(11.6, 12.2)	(15.5, 16.0)
SR (g∙m^−2^∙h^−1^)	78	87	117	90	91	85
	(52, 103)	(58, 116)	(52, 182)	(62, 118)	(59, 122)	(51, 119)
$\:{\stackrel{-}{\text{T}}}_{\text{s}\text{k}}$(°C)	35.6	36.3	36.6	37.2	37.4	36.4
	(35.4, 35.9)	(36.1, 36.6)	(36.3, 36.9)	(37.0, 37.4)	(37.0, 37.8)	(35.8, 37.0)
(R + C) (W∙m^−2^)	−11	−1	9	18	34	24
	(−9, −13)	(−3, 1)	(7, 11)	(16, 20)	(24, 44)	(17, 31)
E_req_ (W∙m^−2^)	66	75	88	103	111	98
	(61, 70)	(69, 81)	(82, 95)	(95, 111)	(99, 124)	(84, 111)
E_max_ (W∙m^−2^)	154	152	171	219	281	227
	(127, 180)	(134, 170)	(152, 190)	(195, 242)	(273, 290)	(219, 235)
ω_max_	0.47	0.52	0.52	0.49	0.39	0.43
	(0.37, 0.57)	(0.45, 0.59)	(0.47, 0.62)	(0.42, 0.55)	(0.36, 0.43)	(0.37, 0.48)

Data are presented as MEAN (95% confidence intervals). T_db_, ambient dry-bulb temperature at critical environmental limit; rh, relative humidity at critical environmental limit; T_wb_, ambient wet-bulb temperature at critical environmental limit; P_a_, ambient water vapor pressure at critical environmental limit; $\:{\stackrel{-}{\text{T}}}_{\text{s}\text{k}}$, mean skin temperature at critical environmental limit; SR; sweat rate; (R + C), radiative and convective heat exchange; E_req_, evaporative cooling required to maintain thermal balance; E_max_, maximal evaporative heat loss; ω_max_, maximal skin wettedness

## Results

Subject characteristics are shown in Table 1. V̇O_2max_ was significantly lower in older compared to young adults (*P* < 0.001), but there were no differences in height, weight, body surface area (A_D_), or body surface area-to-mass ratio (A_D_·kg^−1^) between young and older adults (all *P* ≥ 0.05).

Tables 2 and 3 present environmental conditions, partitional calorimetry parameters, and ω_max_ for young and older adults, respectively, at each metabolic rate for all environmental conditions. There was a main effect of the skin temperature and sweat rate for older compared to young adults during MinAct such that skin temperature and sweat rate are lower in the older cohort (both *P* < 0.05).

**Fig. 1:**
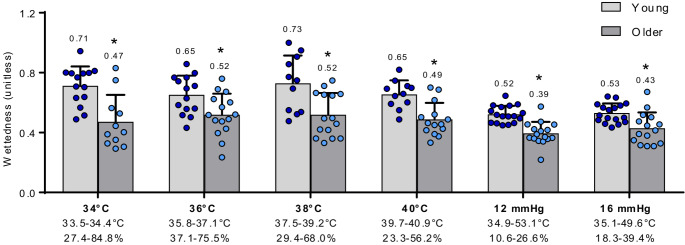
Comparisons of maximal skin wettedness (ω_max_) between young and older adults during minimal activities of daily living. Across all environmental conditions, ω_max_ was lower in older adults compared to their young counterparts (all *P* ≤ 0.008). Filled bars represent means and error bars represent standard deviations. The range of dry-bulb temperature and relative humidity in each environment is placed under the label for each environment. See Table 2 for additional psychometric parameters associated with x-axis values

**Fig. 2:**
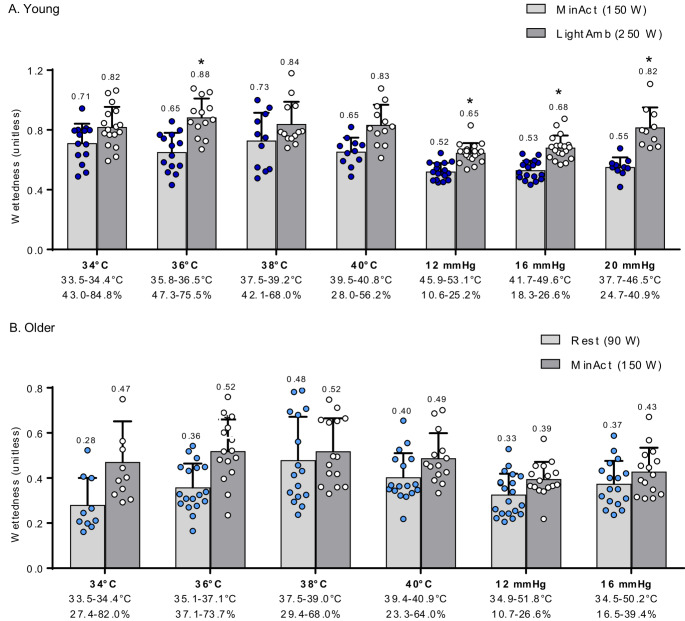
Comparison of maximal skin wettedness (ω_max_) between metabolic rates in young (**A**) and older (**B**) adults. Young adults had lower ω_max_ during minimal activities of daily living (MinAct) compared to light ambulation (LightAmb) in all critical temperature trials (T_crit_; all *P* < 0.007) and the critical water vapor pressure trial (P_crit_) in which temperature was held constant at 36 °C (*P* < 0.007). Older adults had lower ω_max_ at rest compared to MinAct only in the P_crit_ trial in which temperature was held constant at 36 °C (*P* < 0.008). Filled bars represent means and error bars represent standard deviations. The range of dry-bulb temperature and relative humidity in each environment is placed under the label for each environment. See Table 2 for additional psychometric parameters associated with x-axis values

Older adults exhibited lower ω_max_ compared to young adults in all environments during MinAct (all *P* ≤ 0.008; Fig. 1). Young adults had lower ω_max_ during MinAct compared to LightAmb in all T_crit_ trials (all *P* ≤ 0.00002; Fig. 2) and the P_crit_ trial in which T_db_ was held constant at 36 °C (*P* = 0.00007; Fig. 2). Older adults exhibited lower ω_max_ at rest compared to Y during MinAct only in the P_crit_ trial in which T_db_ was held constant at 36 °C (*P* = 0.0008; Fig. 2).

## Discussion

The overall aim of this study was to characterize ω_max_ in unacclimated young and older adults across a wide range of environments and at various low metabolic rates. These data illustrate that ω_max_ in adults over 65 yrs is lower for a given metabolic rate across a wide spectrum of hot and humid environments compared to their young counterparts. Additionally, this study established ω_max_ during light ambulatory activity in young adults and resting older adults in environments ranging from 34 to 49 °C T_db_ and 14–80% rh. These values can be used to model responses to heat stress in these unacclimated populations at a low metabolic rate and potentially aid in the prediction of risk of heat strain.

We have previously reported that ω_max_ decreases with advancing age from 18 to 89 yrs in both a warm-humid and a hot-dry environment during minimal activity (Fisher et al. 2024). Similarly, in the present study across a much larger range of environments from very hot-humid to very hot-dry, ω_max_ was lower in older adults compared to young adults during minimal activities of daily living (Fig. 1). Skin wettedness depends on both the capacity for sweat production as well as the environmental capacity for evaporation (Ravanelli et al. 2018; Mochida et al. 1997). Therefore, lower sweating output coupled with high ambient humidity at the critical environmental limits in older adults may result in a lower proportion of the skin covered with sweat (Tables 2 and 3). Further, higher ambient temperature and/or lower ambient water vapor pressure resulted in lower ω_max_ (Mochida et al. 1997; Atmaca and Yigit 2006), illustrating the dependance of ω_max_ on sweating output as well as environmental conditions.

Further, these data demonstrate variability in ω_max_ as a function of metabolic rate, even the rest-to-low metabolic rates tested herein. For example, in the hot-dry environmental conditions, young adults exhibit higher ω_max_ during LightAmb compared to MinAct (Fig. 2). Higher exercise intensity and therefore metabolic heat production elicits greater sweat rates leading to higher ω_max_ (Ravanelli et al. 2018; Shapiro et al. 1982). Conversely, the elevation in ω_max_ at the higher relative metabolic rate was less evident in the older cohort (Fig. 2). This may be due to a basement effect, given the already low ω_max_ during MinAct in the older cohort.

If known, ω_max_ can be useful in calculating and/or predicting heat balance and related variables. Incorporating ω_max_ values established herein and elsewhere (Ravanelli et al. 2018; Fisher et al. 2024), appropriate for the specific environment, metabolic rate, and cohort, may result in more accurate representation of evaporative heat loss and heat balance. For example, using the following equation evaporative heat loss from the skin in W·m^−2^ can be calculated based on ω and vapor pressure differences between the body and the surrounding air (Atmaca and Yigit 2006).

8 $$\:{E}_{sk}=\frac{\omega\:({P}_{sk}-{P}_{a})}{{R}_{e,t}}$$

Current models using ω_max_ to calculate and/or predict heat exchange between an individual and his/her environment rely on ω_max_ values obtained from seminal work by Candas et al. who reported that highest achievable ω_max_ for unacclimated individuals was 0.85 and 1.00 for heat-acclimated individuals (Candas et al. 1979). During minimal activity in the present study, in some cases ω_max_ was as low as ~ 0.42 and ~ 0.23 and as high as ~ 0.99 and ~ 0.83, in young and older adults respectively, depending on age and environmental conditions. Therefore, sweating capacity, which changes with aging, and environmental temperature and absolute humidity greatly impact ω_max;_ single static values do not accurately represent that variability in ω_max_.

The ω_max_ values established herein may be incorporated into heat stress models to more accurately represent responses in these age cohorts across a wide range of environments and at various metabolic rates, assuming individuals are at their limit of compensable/uncompensable heat stress. However, these ω_max_ values are specific to the cohort, environment, and metabolic rate they were determined in. Additionally, as these calculations are based on previously published data, we are limited in the activities performed by age cohorts during the experiments. Therefore, future research is needed to further determine ω_max_, such as at different activities, higher metabolic rates, and following training and acclimation in older adults.

## Data Availability

Source data supporting the conclusions in this paper are publicly available and can be found at 10.5281/zenodo.14592713.
